# Antidotal Effects of the Antihistamine Diphenhydramine Against Cholinesterase Inhibitor Poisoning: A Meta-Analysis of Median Lethal Doses in Experimental Animals

**DOI:** 10.7759/cureus.54403

**Published:** 2024-02-18

**Authors:** Fouad K Mohammad, Ammar A Mohammed, Ghada A Faris, Banan Al-Baggou, Yaareb J Mousa

**Affiliations:** 1 Department of Physiology, Biochemistry and Pharmacology, College of Veterinary Medicine, University of Mosul, Mosul, IRQ; 2 College of Nursing, The American University of Kurdistan, Duhok, IRQ; 3 Department of Pharmacology, College of Pharmacy, University of Duhok, Duhok, IRQ; 4 Department of Toxicology, College of Veterinary Medicine, University of Mosul, Mosul, IRQ

**Keywords:** cholinergic poisoning, ectoparasiticides, ld50, imidocarb, carbamate, organophosphate, insecticides, antihistamine, antidote, acetylcholinesterase

## Abstract

The H_1_-antihistamine diphenhydramine antagonizes cholinesterase inhibitor poisoning in various animal species. One aspect of acute antidotal actions of diphenhydramine is increasing the median lethal doses (LD50) of toxicants. The objective of this meta-analysis was to assess the antidotal action of diphenhydramine against short-term toxicity (LD50) of cholinesterase inhibitors in experimental animals. The experimental studies selected were according to the Preferred Reporting Items for Systematic Reviews and Meta-Analysis (PRISMA) guidelines. They were conducted in laboratory animals (mice, rats, and chicks) to determine acute LD50 values of cholinesterase inhibitors (organophosphates, carbamates, and imidocarb) under the influence of diphenhydramine vs. controls. Twenty-eight records were selected from 12 studies on mice (n= 242), rats (n= 27), and young chicks (n= 128). The forest plot of randomized two-group meta-analysis assessed effect size, subgroup analysis, drapery prediction, heterogeneity, publication bias-funnel plot as well as one-group proportions meta-analysis of percent protection. Diphenhydramine significantly increased the combined effect size (i.e. increased LD50) in intoxicated experimental animals in comparison to controls (-3.71, standard error (SE) 0.36, 95%CI -4.46, -2.97). The drapery plot proposed a wide range of confidence intervals. The I^2^ index of heterogeneity of the combined effect size was high at 81.03% (*Q*= 142.3, p < 0.0001). Galbraith regression also indicated data heterogeneity; however, the normal quantile plot indicated no outliers. Subgroup analysis indicated significantly high heterogeneity with organophosphates (I^2 ^= 63.72%) and carbamates (I^2 ^= 76.41%), but low with imidocarb (I^2^ = 51.48%). The funnel plot and Egger regression test (t= -13.7, p < 0.0001) revealed publication bias. The median of the diphenhydramine protection ratio was 1.655, and the related forest plot of one group proportion meta-analysis revealed a statistically high level of protection (0.594, SE 0.083, 95%CI 0.432, 0.756), with high heterogeneity (I^2^= 99.86). The risk of bias assessment was unclear, while the total score (16 out of 20) of each study leaned towards the side of the low risk of bias. In conclusion, the meta-analysis of LD50 values indicated that diphenhydramine unequivocally protected experimental animals from the acute toxicity of cholinesterase inhibitors. The drug could be an additional antidote against acute poisoning induced by cholinesterase inhibitors, but a word of caution: it is not to be considered as a replacement for the standard antidote atropine sulfate. Further studies are needed to examine the action of diphenhydramine on adverse chronic effects of cholinesterase inhibitors.

## Introduction and background

Numerous cholinesterase (ChE) inhibiting compounds are available for clinical uses in veterinary medicine as organophosphate and carbamate insecticides, anthelmintics (coumaphos, dichlorvos), and antiprotozoal urea derivatives such as imidocarb [1-3]. Organophosphate and carbamate pesticides are also used in agriculture and public health [4,5]. These medications and agricultural pesticides pose public health hazards, environmental concerns [4,5], and even occupational health hazards among practicing veterinarians or technicians [6]. The single most important mechanism of acute toxic action of these ChE inhibitors is the inhibition of ChE at the neuronal endings, causing accumulation of acetylcholine at synapses and subsequently producing cholinergic toxidrome, which is characterized by nicotinic, muscarinic, and central nervous system effects [7,8]. Atropine sulfate is the standard antidote against poisoning induced by ChE inhibitors, as it blocks muscarinic cholinergic receptors [8,9]. Other antidotal agents such as the ChE reactivators oximes and benzodiazepines have been used too [8,9].

The H1 antihistamine diphenhydramine, with its potent antimuscarinic action, has been applied clinically in human beings and animals to antagonize organophosphate or carbamate poisoning in a manner similar to the standard antidote atropine [10-13]. The antagonistic actions of diphenhydramine against organophosphate or carbamate toxidrome of poisoning and lethality have been documented in experimental laboratory animals such as mice [14-21], rats [19,22-25], and young chicks [26-29]. Additionally, one study reported the antidotal effect of diphenhydramine against imidocarb toxicosis in chicks [30]. A few review articles have delineated the antidotal properties of diphenhydramine against ChE inhibitors [10-13]. These review studies have focused either on the antidotal clinical use of diphenhydramine [10,11], ameliorative action on the cholinergic toxidrome in experimental animals [12], or the decrease in blood and brain ChE inhibition caused by organophosphates and carbamates [13], and one of the recent meta-analyses on diphenhydramine effects has demonstrated that this antihistamine significantly reduces the relative risks of organophosphate and carbamate pesticides by considering signs of poisoning and lethality in experimental laboratory animals [12]. However, none of the reviews cited above have considered analysis of the acute toxicity (median lethal dose, LD50) outcome of ChE inhibitors by the antidotal action of diphenhydramine [10-13], and we took this information gap for further meta-analytic exploration.

Determination of LD50 values is an important index of acute toxicity and lethality of chemicals induced within 24 hours in experimental animals [31]. Therefore, the purpose of the present meta-analysis was to assess an unexplored aspect of the antidotal action of diphenhydramine against short-term toxicity of ChE inhibitors, by identifying studies reporting LD50 values of the latter agents in experimental laboratory animals and demonstrating statistically unequivocal value of diphenhydramine as an additional antidote, but this is not a replacement for the standard one atropine sulfate.

## Review

Methods and data extraction

This meta-analysis was conducted from January 2, 2024, to February 2, 2024, at the College of Veterinary Medicine, University of Mosul, Iraq, and the College of Pharmacy, University of Duhok, Iraq. It was approved by the Reviewing Board at the College of Pharmacy, University of Duhok according to institutional ethics for biomedical research. It was registered on January 28, 2024, at the Center for Open Science (OSF, 0.0B, https://osf.io/), Charlottes, Virginia, United States. The use of experimental animals in all the studies included in the present meta-analysis complied with institutional regulations and ethics, as well as with guidelines of Animal Research: Reporting of In Vivo Experiments (ARRIVE) [32], and the Guide for the Care and Use of Laboratory Animals [33]. 

Search Strategy

We searched for articles and academic theses published from January 1980 to January 2024 on the use of diphenhydramine to affect LD50 values of ChE inhibitors in laboratory experimental animals, using PubMed, Google Scholar, Scopus (Science Direct), Directory of Open Access Journals (DOAJ), Web of Science, My Research Assistant, and Iraqi Academic Scientific Journals (IACSJ) databases. The search strategy included the following search strings across all the databases: “diphenhydramine” “H1 antihistamine”) + “organophosphate”, or “carbamate”, or “imidocarb” “toxicity” or “lethality”; “LD50 of organophosphates” or “carbamates” or “imidocarb” with “diphenhydramine”; “Diphenhydramine antidote against cholinesterase inhibitors” (organophosphates or carbamates or imidocarb). Additionally, we manually searched citations of each published article on the subject of the present study. Articles and academic theses of Iraqi universities that were not listed/indexed in the above-mentioned databases were searched manually. The search strategy did not include any language restriction.

Inclusion Criteria

**Figure 1:**
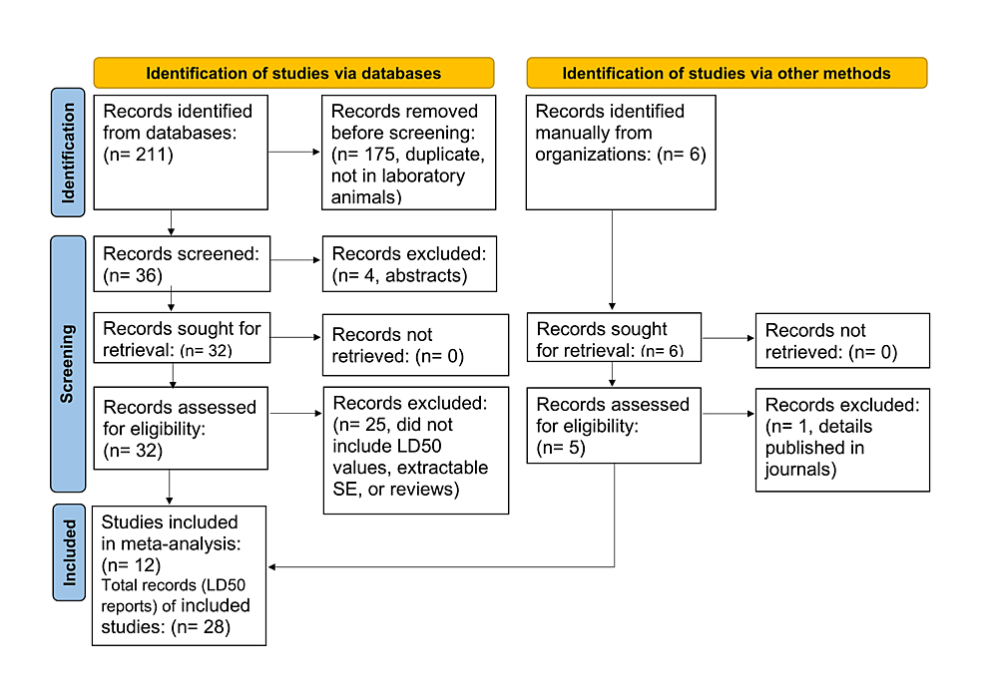
PRISMA flow diagram of the search in databases and other sources reporting median lethal doses (LD50) in experimental animals intoxicated with cholinesterase inhibitors (organophosphates, carbamates and imidocarb), and treated with diphenhydramine. PRISMA: Preferred Reporting Items for Systematic Reviews and Meta-Analysis

As shown in Figure 1, the selection of studies for the present meta-analysis was according to the Preferred Reporting Items for Systematic Reviews and Meta-Analysis (PRISMA) [34]. Criteria for the selection of studies were based on experimental studies conducted to determine acute-24 h LD50 values of ChE inhibitors under the influence of diphenhydramine (experimental intervention) with proper controls (no-diphenhydramine). The administration of diphenhydramine was either before or after the ChE inhibitor intoxication. ChE-inhibitors considered for the present meta-analysis were those with established anti-ChE action from toxicological/pharmacological points of view. The final selection consisted of 12 studies [16-22,26-30], that included 28 records of changes (or no change) in LD50 values induced by diphenhydramine in experimental animals treated with ChE inhibitors (Figure 1, Table 1). Some of the 12 studies included more than one set of LD50 experiments; therefore, the final records included 28 LD50 experimental results. All authors were involved in the study selections.

**Table 1:** Effect of diphenhydramine on the median lethal dose (LD50) of cholinesterase inhibitors in experimental animals *Two citations (the first one is a thesis) for records are complimentary to each other for complete presentation of the parameters and treatments information. Protection ratio= LD50 with diphenhydramine/LD50 control ChEI: cholinesterase inhibitor; sc: subcutaneous injection; ip: intraperitoneal injection; im: intramuscular injection; Diph: diphenhydramine, n: number of animals. Records were coded from A to Zb for the convenience of presentation in meta-analysis.

Authors and year*	Code	Animal species	ChEI, route of administration	n	LD50-Control	SE	95% CI	n	LD50+Diph	SE	95% CI	Protection ratio	Diph treatments
Faris, 1995 [16]; Faris and Mohammad, 1997 [17]	A	Mice	Dichlorvos, orally	7	79.2	10.91	68.29, 90.11	8	104.2	12.94	91.26, 117.14	1.32	Diph 30 mg/kg, sc 15min before ChEI
Faris, 1995 [16]; Faris and Mohammad, 1997 [17]	B	Mice	Dichlorvos, orally	7	79.2	10.91	68.29, 90.11	11	145.8	15.68	130.12, 161.48	1.84	Diph 30 mg/kg, sc 15 min before ChEI, repeated 1-3 times during first 90 min
Faris, 1995 [16]; Faris and Mohammad, 1997 [17]	C	Mice	Dichlorvos, orally	7	79.2	10.91	68.29, 90.11	10	120.8	13.39	107.41, 134.19	1.53	Diph 30 mg/kg, sc immediately after dichlorvos dosing, repeated 1-3 times during first 90 min
Faris, 1995 [16]; Faris and Mohammad, 1997 [17]	D	Mice	Dichlorvos, orally	7	79.2	10.91	68.29, 90.11	12	131.3	14.76	116.54, 146.06	1.66	Diph 30 mg/kg, ip immediately after dichlorvos dosing, repeated 1-3 times during first 90 min
Faris, 1995 [16]; Faris and Mohammad, 1996 [18]	E	Mice	Diazinon, orally	7	76	6.26	69.74, 82.26	11	130	12.55	117.45, 142.55	1.71	Diph 20 mg/kg, sc 5 min after diazinon dosing
Faris, 1995 [16]; Faris and Mohammad, 1996 [18]	F	Mice	Methidathion, orally	7	10.5	1.11	9.39, 11.61	9	22.5	1.89	20.61, 24.39	2.14	Diph 20 mg/kg, sc 5 min after methidathion dosing
Faris, 1995 [16]; Faris and Mohammad, 1996 [18]	G	Mice	Malathion, orally	5	650	52.92	597.08, 702.92	9	1050	74.53	975.47, 1124.53	1.62	Diph 20 mg/kg, sc 5 min after malathion dosing
Faris, 1995 [16]; Faris and Mohammad, 1996 [18]	H	Mice	Fenitrothion, orally	10	917	74.39	842.61, 991.39	6	1150	84.98	1065.02, 1234.98	1.25	Diph 20 mg/kg, sc 5 min after fenitrothion dosing
Al-Baggou 1997 [19]; Al-Baggou and Mohammad, 1998 [20]	I	Mice	Physostigmine, sc	5	0.673	0.11	0.563, 0.783	10	2.295	0.236	2.059, 2.531	3.41	Diph 10 mg/kg, sc 15 min. before ChEI
Al-Baggou 1997 [19]; Al-Baggou and Mohammad, 1998 [20]	J	Mice	Physostigmine, sc	5	0.673	0.11	0.563, 0.783	6	3.710	1.514	2.196, 5.224	5.51	Diph 20 mg/kg, sc 15 min. before ChEI
Al-Baggou 1997 [19]; Al-Baggou and Mohammad, 1998 [20]	K	Mice	Neostigmine, sc	6	0.785	0.151	0.634, 0.936	7	1.435	0.143	1.292, 1.578	1.83	Diph 15 mg/kg, sc 15 min. before ChEI
Al-Baggou 1997 [19]; Al-Baggou and Mohammad, 1998 [20]	L	Mice	Neostigmine s.c	6	0.785	0.151	0.634, 0.936	5	1.021	0.087	0.934, 1.108	1.30	Diph 20 mg/kg, sc 15 min. before ChEI
Al-Baggou 1997 [19]; Al-Baggou and Mohammad, 1998 [20]	M	Mice	Neostigmine, sc	6	0.785	0.151	0.634, 0.936	5	0.925	0.087	0.838, 1.012	1.18	Diph 30 mg/kg, sc 15 min. before ChEI
Al-Baggou 1997 [19]; Al-Baggou' and Mohammad, 1999 [22]	N	Rats	Methomyl, orally	7	6.29	1.0434	5.2466, 7.3334	6	10.74	0.87	9.87, 11.61	1.71	Diph 10 mg/kg, sc with ChEI
Al-Baggou 1997 [19]; Al-Baggou' and Mohammad, 1999 [22]	O	Rats	Methomyl, orally	7	6.29	1.0434	5.2466, 7.3334	7	11.00	1.205	9.795, 12.205	1.75	Diph 20 mg/kg, sc with ChEI
Al-Shammary, 2008 [26]	P	Chicks	Diazinon, orally	6	5.7	1.21	4.49, 6.91	6	10.3	1.21	9.09, 11.51	1.81	Diph 10 mg/kg, im 15 min before ChEI
Al-Shammary, 2008 [26]	Q	Chicks	Chlorpyrifos, orally	8	27.4	2.5	24.90, 29.91	10	45.2	2.83	42.37, 48.03	1.65	Diph 10 mg/kg, im 15 min before ChEI
Al-Shammary, 2008 [26]	R	Chicks	Methidathion, orally	9	35.4	2.83	32.57, 38.23	10	54.4	3.13	51.27, 57.53	1.54	Diph 10 mg/kg, im 15 min before ChEI
Mousa and Mohammad, 2009 [27]	S	Chicks	Dichlorvos, orally	5	15.2	1.04	14.16, 16.24	8	26.2	2.28	23.92, 28.48	1.72	Diph 10 mg/kg, im 15 min before ChEI
Ibraheem, 2011 [30]	T	Chicks	Imidocarb, sc	7	98.19	5.71	92.48, 103.90	6	103.47	0.87	102.60, 104.34	1.05	Diph 5 mg/kg, sc 5 min before ChEI
Ibraheem, 2011 [30]	U	Chicks	Imidocarb, sc	7	98.19	5.71	92.48, 103.90	8	104.36	1.42	102.94, 105.78	1.06	Diph 5 mg/kg, sc immediately after ChEI
Ibraheem, 2011 [30]	V	Chicks	Imidocarb, sc	7	98.19	5.71	92.48, 103.90	6	98.20	1.21	96.99, 99.41	0.00	Diph 10 mg/kg, im immediately after ChEI
Mohammad et al., 2012 [28]	W	Chicks	Dichlorvos, orally	6	6.49	1.3	5.19, 7.79	7	17.14	1.57	15.57, 18.71	2.64	Diph 10 mg/kg, im immediately after ChEI
Al-gargary, 2022 [21]	X	Mice	Dichlorvos , orally	5	57.67	11.18	46.49, 68.85	6	95.78	10.81	84.97, 106.59	1.66	Diph 10 mg/kg, sc 15 min. before ChEI
Al-gargary, 2022 [21]	Y	Mice	Dichlorvos, orally	5	57.67	11.18	46.49, 68.85	6	95.78	10.87	84.91, 106.65	1.66	Diph 20 mg/kg, sc 15 min. before ChEI
Al-gargary, 2022 [21]	Z	Mice	Carbaryl, orally	5	824.4	105.42	718.98, 929.82	8	1309	83.45	1225.55, 1392.45	1.59	Dose of Diph 10 mg/kg, sc 15 min. before ChEI
Al-gargary, 2022 [21]	Za	Mice	Carbaryl, orally	5	824.4	105.42	718.98, 929.82	8	1309	83.45	1225.55, 1392.45	1.59	Diph 20 mg/kg, sc 15 min. before ChEI
Mohammed and Mohammad, 2022 [29]	Zb	Chicks	Carbaryl, orally	7	207	26.1	180.9, 233.1	5	335	17.32	317.68, 352.32	1.62	Diph 10 mg/kg, im 15 min before ChEI

Exclusion Criteria

We excluded from the present meta-analysis experimental studies using other antidotes or non-diphenhydramine antihistamines, studies not reporting LD50 values of ChE inhibitors, or those that did not have enough data to extract the related statistics of LD50 values, such as the number of animals used, the standard deviation, 95% confidence interval (CI), or the standard error (SE). Studies using non-ChE inhibitors were not included in the meta-analysis. Duplicate records/abstracts were also excluded from the meta-analysis.

Data Extraction

The 24-hour acute LD50 values of ChE inhibitors with diphenhydramine as the intervention group and control groups (without diphenhydramine) as described in the selection criteria were extracted from texts, tables, or figures of the selected studies. Some studies reported more than one subset of data on the related LD50 values of ChE inhibitors, and these were considered as individual entities of LD50 records which were included in the meta-analysis (Table 1). All studies of the present meta-analysis have used the up-and-down method to determine the LD50 value [35] in accordance with the ARRIVE guidelines to minimize the number of experimental animals (usually < 10 for each LD50 determination). Each LD50 value of a particular ChE inhibitor with or without diphenhydramine was determined using the formula of Dixon [35]: LD50= xf + kd, where xf is the last dose of the ChE inhibitor administered, d is the increase or decrease in the ChE inhibitor dose and k is a value from the table of Dixon [35] with an SE of 0.61. We then calculated the SE and 95% CI of each LD50 value as outlined earlier [36,37]. For the final meta-analysis, we tabulated the results as records obtained from studies that included the number of experimental animals used, LD50 values with and without diphenhydramine, SE, 95% CI, and diphenhydramine antidotal protocols. Further, the protection ratio was estimated as follows: Protection Ratio= LD50 of ChE inhibitor with diphenhydramine/LD50 of ChE inhibitor without diphenhydramine. Protection ratios were also tabulated for another meta-analysis to present the forest plot of percentages (0-100%) of protection via diphenhydramine (Table 2).

**Table 2:** Diphenhydramine protection of experimental animals intoxicated with cholinesterase inhibitors *Protection ratio= LD50 with diphenhydramine / LD50 control ** Maximum protection was considered 100% for the purpose of one-group proportional meta-analysis. Citations of record codes are presented in Table 1.

Record code	Animal species	Protection ratio*	% protection**
A	Mice	1.32	32
B	Mice	1.84	84
C	Mice	1.53	53
D	Mice	1.66	66
E	Mice	1.71	71
F	Mice	2.14	100
G	Mice	1.62	62
H	Mice	1.25	25
I	Mice	3.41	100
J	Mice	5.51	100
K	Mice	1.83	83
L	Mice	1.30	30
M	Mice	1.18	18
N	Rats	1.71	71
O	Rats	1.75	75
P	Chicks	1.81	81
Q	Chicks	1.65	65
R	Chicks	1.54	54
S	Chicks	1.72	72
T	Chicks	1.05	5
U	Chicks	1.06	6
V	Chicks	0.00	0
W	Chicks	2.64	100
X	Mice	1.66	66
Y	Mice	1.66	66
Z	Mice	1.59	59
Za	Mice	1.59	59
Zb	Chicks	1.62	62
Mean		1.763	-
SE		0.175	
SD		0.924	-
Median		1.655	-
25 percentile		1.3725	-
75 percentile		1.795	-

All authors of the present review were involved in the literature search, data acquisition, and calculation of SE, 95% CI, and protection ratios. We resolved any disagreement regarding the inclusion or exclusion of studies, data extraction, and related calculations by consensus, and agreement was achieved with the reviewer FKM.

Statistical Analysis

Considering PRISMA guidelines, we applied a two-arm meta-analysis (diphenhydramine as antidote vs. control-no diphenhydramine) using the random effects model to accommodate expected sources of heterogeneity among different studies which mainly included different ChE inhibitors used, experimental animal species, routes, and times of diphenhydramine administrations relative to toxicant doing (Table 1). To conduct the meta-analysis, we used the software program Meta-Essentials Version 1.5 (Erasmus Research Institute of Management, Rotterdam, Netherlands) [38]. This analysis included the forest plot to calculate the effect size, weighted means with their 95% CI, the weight of each study, and the Z-test values at p < 0.05 as well as the Galbraith, normal quantile plot, and funnel plots [38,39]. Furthermore, the protection ratios of diphenhydramine calculated from records of the studies were statistically analyzed to estimate the mean, SE, SD, median, and 25th and 75th percentiles. Diphenhydramine protection against the ChE inhibitor-induced lethality was also subjected to one-group proportions meta-analysis by considering the percent protection that ranged between 0% (no protection) to 100% (complete protection) using the online software OpenMeta[Analyst] (Brown University, Rhode Island, United States) [40].

Drapery Plot

To further support the meta-analysis results, a drapery plot was constructed with its proposed confidence intervals at a p-value of < 0.05, using the Meta-Mar software (Philipps-Universität Marburg, Marburg, Germany). The plot utilizes functions of p-values of each record, with y-axis = p-values and the x-axis = effect sizes, and it merely points out the prediction range, at a certain significance level such as p < 0.05, of a single study that could be conducted in the future [41].

Heterogeneity Analysis

The heterogeneity of the tabulated records was statistically analyzed by the Cochrane Q-test at a p-value of < 0.10 [42-44]. Additionally, the value of I2 was calculated by the Meta-Essentials program mentioned above. Values of I2 may range from no heterogeneity (0%) to a high level of heterogeneity (100%) [42-44]. We subjected all 28 records of mice, rats, and chicks that were treated with organophosphates (n= 15), carbamates (n= 10), or imidocarb (n=3) to subgroup analysis [38,39,44]. This is because these compounds differ in their ChE inhibitory actions and toxicity potentials.

Publication Bias

The funnel plot, which comprised effect size against the SE, was used to assess visually any publication bias, whereas the Egger statistical test was objectively applied to confirm the existence of any bias [44,45].

Risk of Bias

The risk of bias was evaluated as described earlier for animal studies [46,47]. The risk of bias tool consisted of 10 questions that encompassed six bias aspects, which covered selection (allocation sequence, baseline similarity, and concealed allocation), performance (random housing and blindness to intervention), detection (random selection and blind assessment), attrition, reporting, and other interfering problems) [46]. Answers of “yes”, “no” and “unclear” indicated low (score of 2), high (score of 0), and moderate (score of 1) bias outcomes, respectively. A moderate (unclear) outcome indicates that one or more sub-questions of a particular bias have been fulfilled partially (score of 1), whereas a high risk of bias is indicated by the no-response (score of 0) to any sub-question [46,47]. The online tool robvis [48] was used to create the overall risk-of-bias assessments.

Results

Data Extraction and Tabulation

Figure 1 shows the PRISMA flow chart for selecting studies that included LD50 records with or without diphenhydramine in experimental animals intoxicated with ChE inhibitors. The initial search retrieved 211 studies which included the use of diphenhydramine as an antidote and/or protective agent against ChE inhibitors. The final list of selections comprised 28 records of LD50 values for meta-analysis (Figure 1, Table 1). These records included LD50 values of ChE inhibitors in three experimental animal species, mice (n= 242), rats (n= 27), and young chicks (n= 128). The total number of diphenhydramine-treated animals was 216, whereas that of the controls was 181 (Table 1). The ChE inhibitors covered by the present meta-analysis included the organophosphate insecticides dichlorvos, diazinon, methidathion, malathion, fenitrothion and chlorpyrifos, the carbamate insecticides methomyl and carbaryl, the carbamate anti-ChEs physostigmine and neostigmine, as well as the anti-parasitic agent imidocarb (Table 1). These studies appeared in the literature between 1995 and 2022. Diphenhydramine ameliorated the acute toxicity of ChE inhibitors by increasing their LD50 values (Table 1) and elevating the protection ratio (0-5.51), which represented 0% protection in one record and 100% protection in the rest of the records (Table 2).

Meta-Analysis

**Figure 2:**
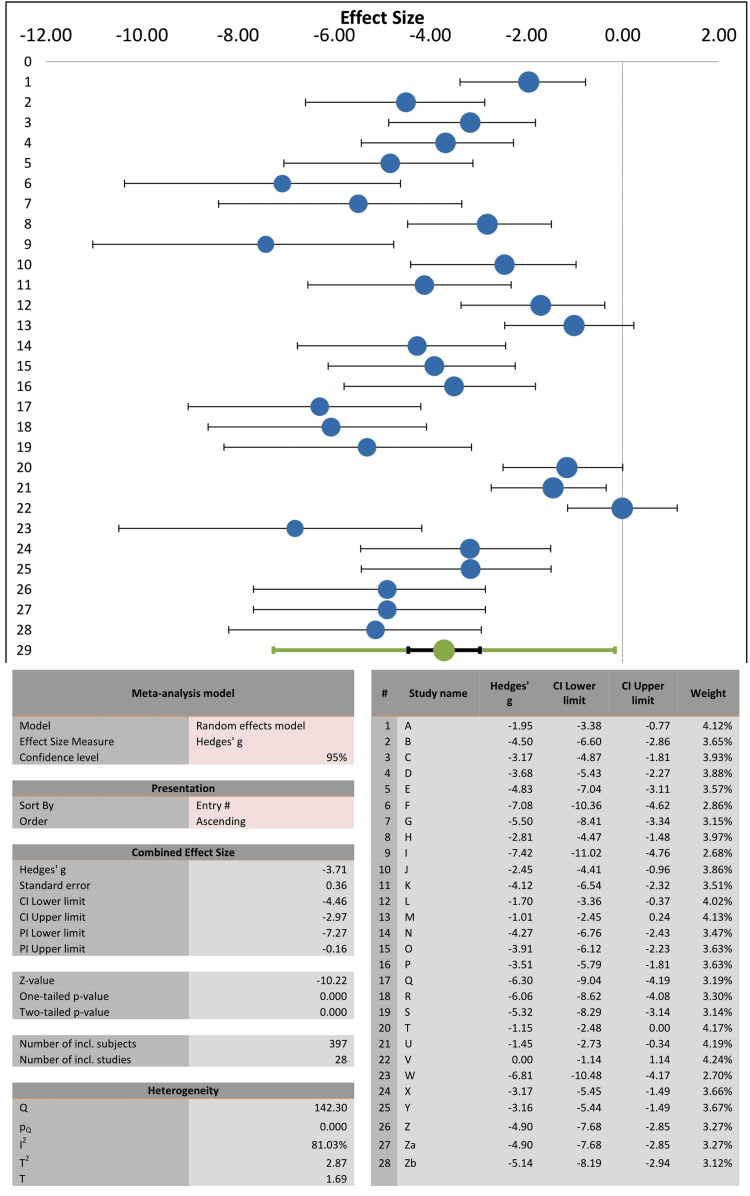
Forest plot of reports comparing median lethal dose (LD50) values of cholinesterase inhibitors (organophosphates, carbamates, and imidocarb) in experimental animals (mice, rats, and chicks) with diphenhydramine treatments (left side of zero effect size) or with untreated-no diphenhydramine controls (right side of zero effect size).

Two-group randomized effects model meta-analysis of the present 28 records of LD50 values (total experimental animals= 397) revealed by the forest plot that the combined effect size (representing increased LD50 values) was significant and more favorable in intoxicated experimental animals treated with diphenhydramine in comparison with respective non-diphenhydramine control groups (Figure 2). The combined effect size of diphenhydramine therapy was -3.71, with an SE of 0.36, 95% C.I. lower limit -4.46, and 95% CI upper limit -2.97, Z value -10.22, and two-tailed p-value= 0.0001 (Figure 2). As shown by the forest plot and its related data, the percentages of weights of study records were close to each other, and they ranged from 2.68% to 4.24%.

Drapery Plot

**Figure 3:**
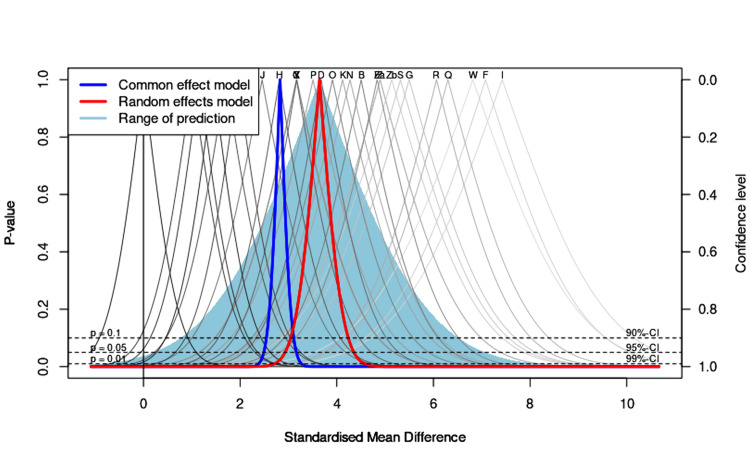
Drapery plot of reports comparing median lethal dose (LD50) values of cholinesterase inhibitors (organophosphates, carbamates, and imidocarb) in experimental animals (mice, rats, and chicks) with diphenhydramine treatments. The confidence curves depict the p-value function of each report and meta-analysis estimates. The shaded area shows a prediction region for a single future study.

Construction of the predictive drapery plot, which is considered a supportive tool for the forest plot, proposed a wide range of CI based on functions of p-values of each record (Figure 3). As shown in Figure 3, the drapery plot with its proposed confidence intervals, supported the present meta-analysis, after considering the p-value function of each record (y-axis= p-values; x-axis= effect sizes).

Heterogeneity Analysis

**Figure 4:**
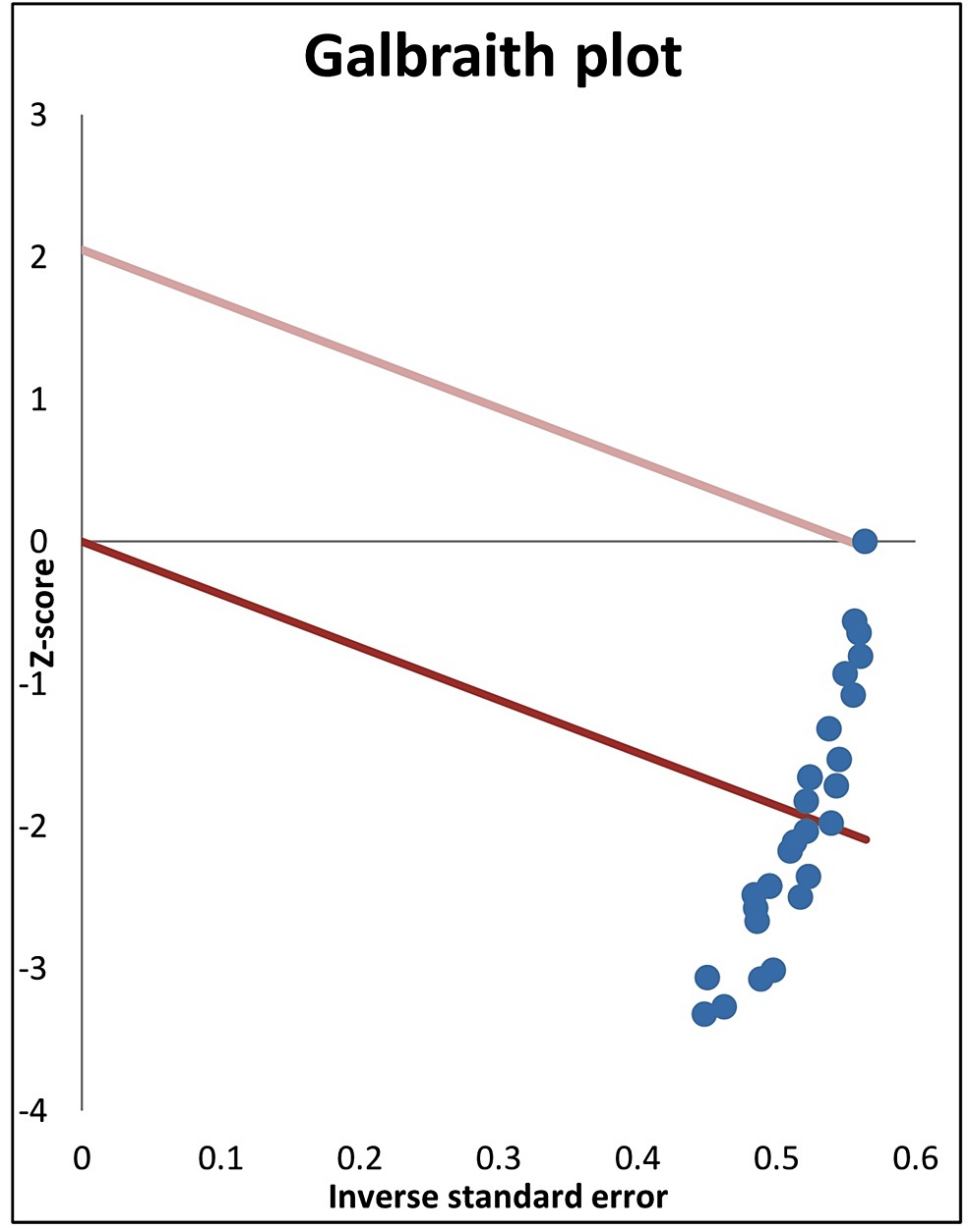
Galbraith plot of median lethal dose (LD50) values (28 records) of cholinesterase inhibitors (organophosphates, carbamates, and imidocarb) in experimental animals (mice, rats, and chicks) with diphenhydramine treatments

**Figure 5:**
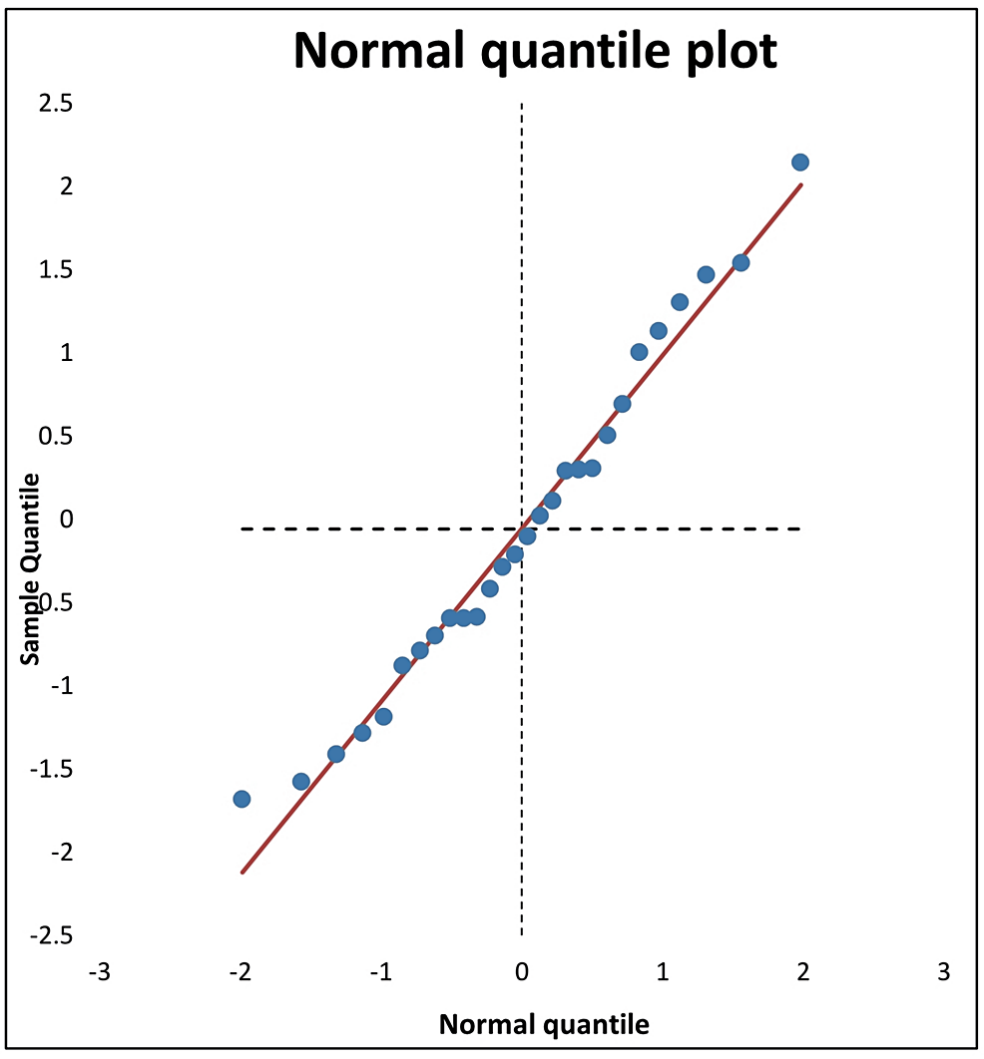
Quantile plot of median lethal dose (LD50) values (28 records) of cholinesterase inhibitors (organophosphates, carbamates, and imidocarb) in experimental animals (mice, rats, and chicks) with diphenhydramine treatments.

Using the Cochrane Q-test and the I2 index, we examined the heterogeneity among the studies of the present random effects model of meta-analysis (Figure 2). The I2 index of heterogeneity of the combined effect size was 81.03%, indicating a high degree of heterogeneity, with T2 and T values of 2.87 and 1.69, respectively. The calculated I2 index may range from 0% (no heterogeneity) to 100% (high heterogeneity). The high level of heterogeneity of the present meta-analysis was also confirmed to be statistically significant using the Cochrane Q-test (Q= 142.3, p < 0.0001). Additionally, Galbraith regression analysis of Z score vs inverse SE (Figure 4) indicated that the data points of effect size were heterogeneous, and in support of the forest plot, they were below the reference no effect line (y = 0). However, despite the heterogeneity of the data, the normal quantile plot indicated no outliers as the data points were mostly limited to the regression line (Figure 5).

Subgroup Analysis

**Figure 6:**
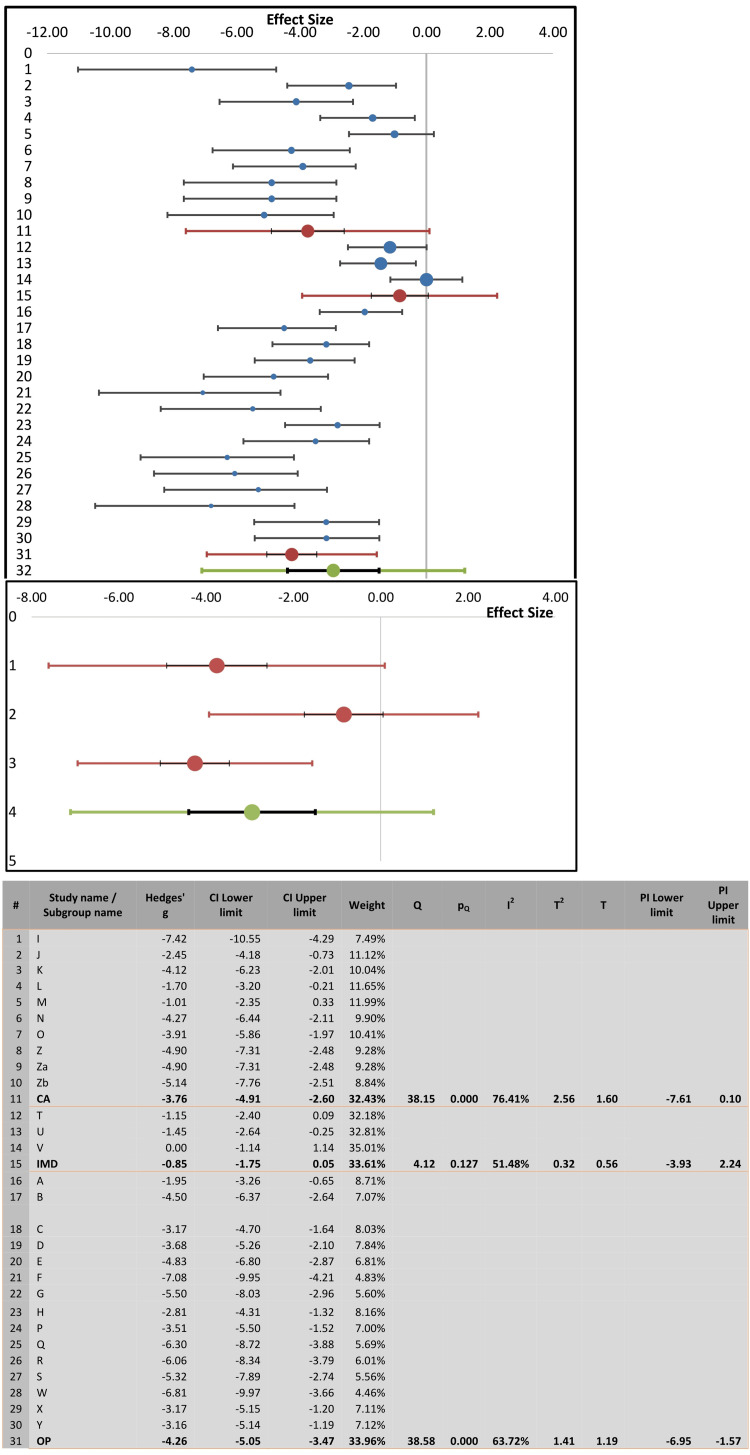
Subgroup analysis and related forest plots to identify heterogeneity of reports comparing median lethal dose (LD50) values of cholinesterase inhibitors (organophosphates, carbamates, and imidocarb) in experimental animals (mice, rats, and chicks) treated with diphenhydramine or not (untreated-no diphenhydramine controls).

To further analyze the cause of the high heterogeneity (I2 = 81.03%), we conducted a subgroup analysis according to the types of toxicants applied in the studies, which were organophosphates, carbamates, or imidocarb in the three experimental animal species (mice, rats, or chicks). Figure 6 shows the forest plot of the subgroup analysis and the related statistical values. The heterogeneity remained significantly high with organophosphates (I2= 63.72%, Q= 38.58, p < 0.0001) and carbamates (I2 = 76.41%, Q= 38.15, p < 0.0001), but low with imidocarb (I2= 51.48%, Q= 4.12, p= 0.127). Correspondingly, the standardized measures of effect size (Hedge’s g) for the three toxicants were -4.26, -3.76, and -0.85, respectively (Figure 6). The related values of T2 and T remained low in the three subgroups, organophosphates (1.41, 1.19), carbamates (2.56, 1.6), and imidocarb (0.32, 0.56). The weights of the three subgroups were 33.96%, 32.43%, and 33.61%, respectively. Additionally, the pseudo-R2 value of the overall subgroup analysis was 57.88%, which is an indication of a relatively moderate heterogeneity. However, from the data presented in Figure 6, most of the heterogeneity could be attributed to organophosphate and carbamate subgroups.

Funnel Plot to Examine the Publication Bias

**Figure 7:**
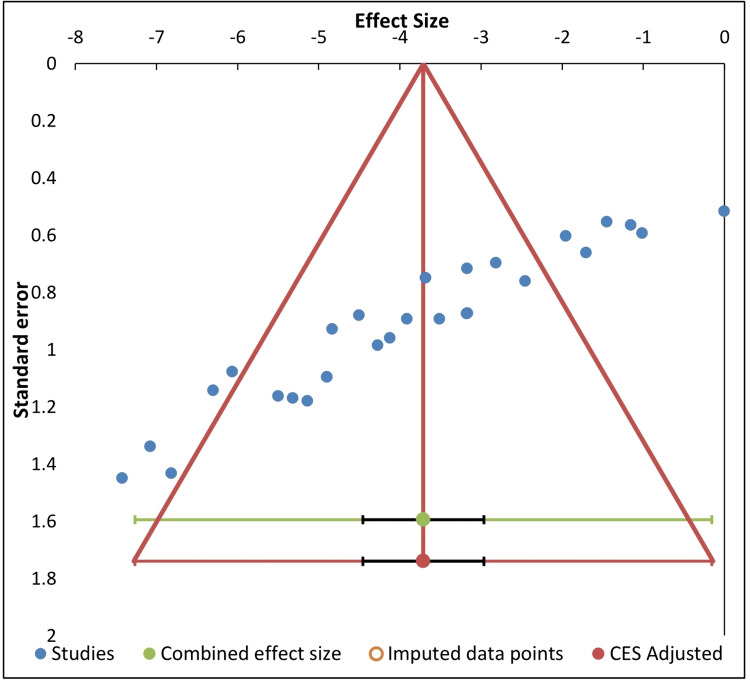
Funnel plot to identify any publication bias

Visual examination of the constructed funnel plot (SE vs effect size) revealed publication bias, as 11 effect size points strayed from both sides of the symmetrical distribution within the limited area of the plot (Figure 7). This interpretation of the publication bias was further objectively and statistically confirmed by the Egger regression test which was significant (t= -13.7, p < 0.0001). However, despite the evidence of publication bias in the present meta-analysis, the trim-and-fill analysis, which is an adjustment for potentially missing records of the funnel plot did not reveal any imputed point to adjust the combined effect size (Figure 7).

**Figure 8:**
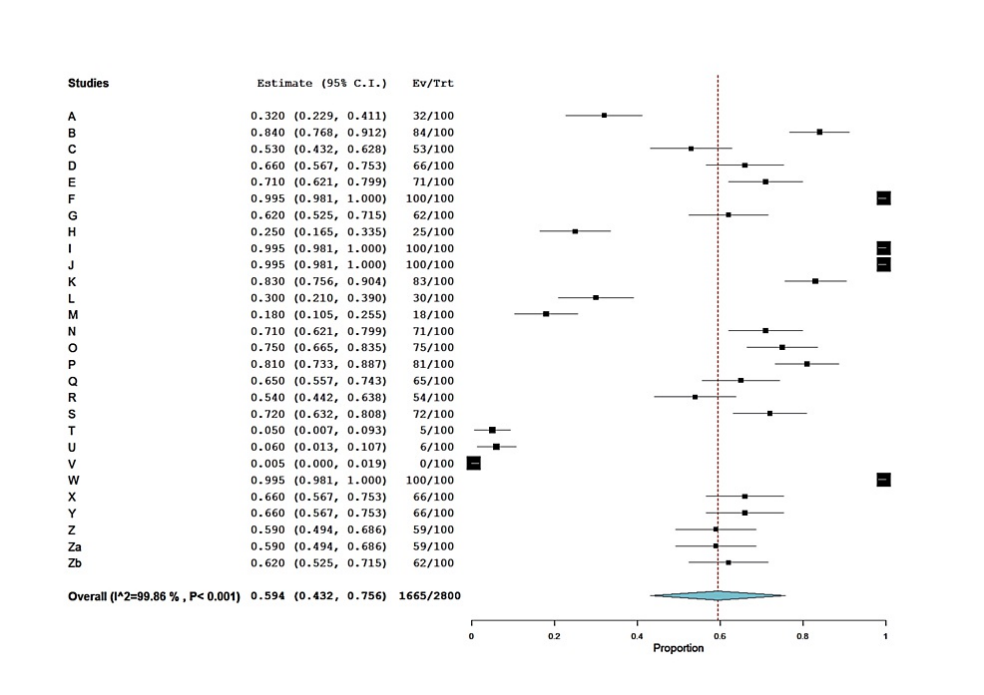
Forest plot of diphenhydramine-induced protection of experimental animals (mice, rats, and chicks) intoxicated with cholinesterase inhibitors (organophosphates, carbamates, and imidocarb). Diphenhydramine treatments are on the right side of zero effect size and the controls are on the left side.

In support of the above-mentioned meta-analysis, an additional analysis of the diphenhydramine protection indicated that the median of the protection ratios (LD50 with diphenhydramine / LD50 without diphenhydramine) was 1.655 with a mean of 1.763 (Table 2). The related forest plot of one group proportion meta-analysis, considering the % protection (0-100%) as the input data, revealed that diphenhydramine therapy indeed manifested a statistically high level of protection (combined effect size= 0.594, SE= 0.083, 95%CI= 0.432, 0.756; p < 0.001), but with a concomitant high level of heterogeneity (I2= 99.86; Q= 18816.8, p < 0.001) (Figure 8).

Risk of Bias

**Figure 9:**
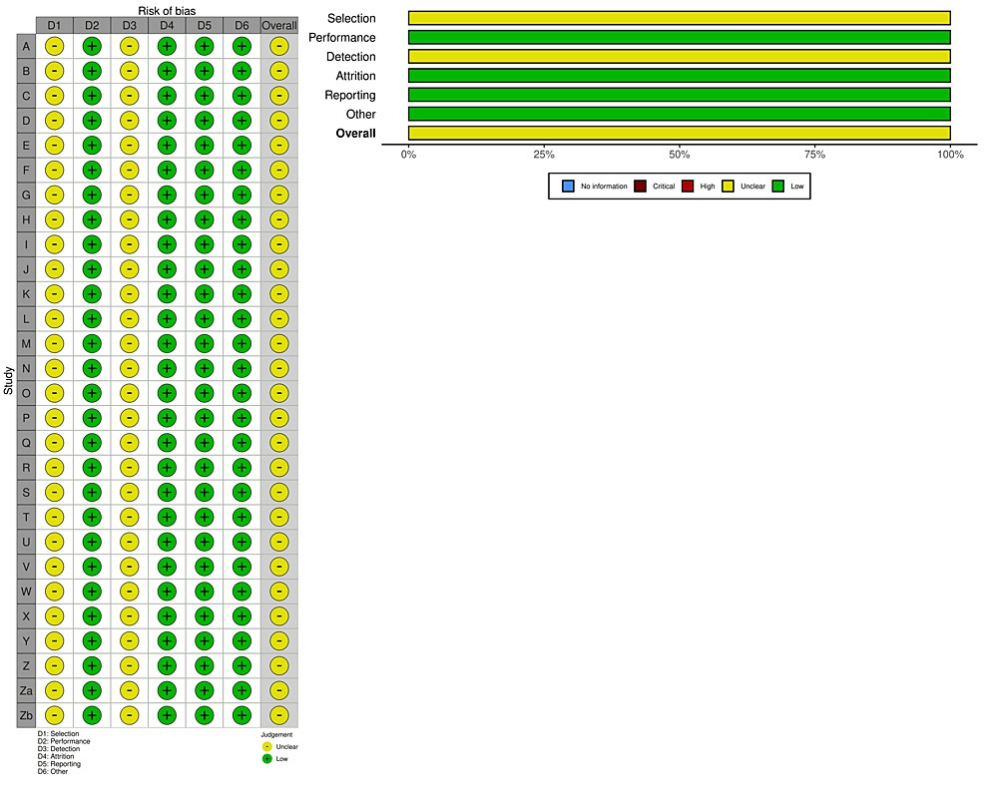
Risk of bias distribution across the 28 study records used for the meta-analysis. The online tool robvis [48] was used to create the figure.

As shown in Figure 9, seven out of 10 items of the 28 study records were assessed as low risk of bias, two were moderate or unclear (selection sub-question on concealed allocation) and one was at high risk of bias (blind assessment). Furthermore, the overall assessment of the risk of bias was “unclear”. However, the total score (16 out of 20) of each study leaned towards the side of the low risk of bias.

Discussion

The present meta-analysis of LD50 values indicated that diphenhydramine unequivocally protected experimental animals (mice, rats, and chicks) from the acute toxicity of ChE inhibitors. This result is in agreement with a recent meta-analysis of the antidotal action of diphenhydramine against organophosphate and carbamate insecticides taking into consideration the acute toxidrome of these insecticides, but not their LD50 values [12]. LD50 values of toxicants are excellent indices of their acute toxicity, especially when conducted within 24 hours [31,36]. However, a major drawback of acute LD50 studies is the overuse of large numbers of experimental animals [31]. Alternative LD50 experiments, mainly the up-and-down method, partially overcome this dilemma by using fewer (usually < 10) animals in each test [35-37]. This was accomplished by all the 12 studies and their 28 reports included in the present meta-analysis. These studies are also in accordance with the ARRIVE, considering the reduction of laboratory animals in toxicity studies.

The mechanism of ameliorative action of diphenhydramine against ChE inhibitors’ intoxication is most probably due to the potent antimuscarinic property of this antihistamine [10-12]. There is also a possibility of additional anti-nicotinic and anti-ChE (weak inhibition) actions of diphenhydramine that might contribute to this antidotal action [12,13]. However, we stress within this context that diphenhydramine is not advocated here as a replacement for the standard antidote atropine, it is merely an additional therapeutic agent that could be beneficial in certain cases of toxicosis by ChE inhibitors. However, the effects of this antihistamine on chronic poisoning induced by ChE inhibitors, especially the insecticides, are not known.

The present meta-analysis shows strong evidence of antidotal action of diphenhydramine against the acute 24-hour toxicity (LD50) of ChE inhibitors. This was indicated by the significant combined effect size of diphenhydramine therapy which was -3.71 (SE= 0.36, 95%CI= -4.46, -2.97), with a median protection ratio of 1.655 (mean= 1.763) and the combined effect size of % protection was 0.594 (SE= 0.083, 95%CI= 0.432, 0.756), whereas weights of study records were close to each other. However, high degrees of heterogeneity were observed using the Cochrane Q-test and the I2 index. This prompted us to analyze the cause of heterogeneity and it was evident from the subgroup analysis that most of the significant heterogeneity was attributed to organophosphate (I2= 63.72%) and carbamate (I2= 76.41%) subgroups; that of the imidocarb was not significant (I2 = 51.48%). That of the imidocarb subgroup could be attributed to the limited number of records (n= 3) in the present analysis. It is also possible that the source of heterogeneity could be associated with the type of ChE inhibitor and its inherent toxicity potential in different laboratory animal species reported by the studies (Table 1). Overall, in support of forest plot heterogeneity results, Galbraith regression analysis of Z score vs inverse SE (Figure 4) indicated heterogeneity of data points of the effect size. Nevertheless, despite this heterogeneity, the normal quantile plot indicated no data outlier.

Visual inspection of the contour-enhanced funnel plot and Egger regression analysis indicated the existence of the publication bias since effect size points surpassed both sides of the symmetrical distribution within the limited area of the plot. Publication bias could result from selective reporting of results that are significant, or it might occur by limiting the study to a selective outcome, or small-level studies [44,45,49]. However, the data points of the present meta-analysis were without outliers as shown by the normal quantile plot, and they were symmetrically distributed in and around the designated area of the funnel plot, with no imputed points or skewness. Within this context of limited publication bias observed, caution is still recommended in interpreting the present LD50 data for clinical application in cases of animal poisoning. To this end, we cautiously draw a conclusion regarding the conduction of additional acute toxicity studies based on LD50 values of ChE inhibitors, since the prediction of future studies by the drapery plot appeared to be of a wide range at p = 0.05. Therefore, additional studies are warranted on the antidotal effects of diphenhydramine, other than increasing the LD50, following various ChE inhibitors intoxications in experimental animals. It is worth mentioning, however, that clinical studies in cats, dogs, or chickens further support the integrity and usefulness of diphenhydramine antidotal therapy against poisoning induced by organophosphate or carbamate insecticides, with no apparent adverse drug-drug interactions [50-52]. Because of the lack of such a drug-drug interaction, clinical studies have used the reversible ChE inhibitor, physostigmine to counteract diphenhydramine anticholinergic toxicity [53].

The risk of bias outcome was not high, but mostly low, since the total risk score of each record included in the present meta-analysis was high (16 out of 20). However, the final assessment of the risk appeared to be inconclusive (unclear). This is because of “unclear” answers given to three sub-questions of the selection (concealed allocation), performance (intervention blindness), and detection (blind assessment) of bias aspects (Figure 9). This overall “unclear” risk assessment could be related to the nature of the up-and-down LD50 determination [35], which uses one experimental animal at a time for each treatment intervention, a condition that precludes blindness to treatments or the outcome assessment; hence, answers to the above-mentioned sub-questions became unsatisfactory.

Limitations of the study

The present meta-analysis was only on the acute toxicity of ChE inhibitors that involved the LD50 values of ChE inhibitors. None of the studies addressed chronic toxicity outcomes. The study included publications generated at Iraqi universities. Other international studies, e.g., a reference on diphenhydramine effects on anti-ChE lethality in experimental animals did not include LD50 values [24]. The unclear rate of the risk of bias could have been due to the nature of the LD50 experiment that uses one animal/treatment/each time point [34]. However, strict adherence to ARRIVE guidelines in studies involving experimental animals would minimize the risk of bias.

## Conclusions

The present meta-analysis of LD50 values indicated that diphenhydramine could be an additional antidote against acute poisoning induced by ChE inhibitors suitably using the three animal species (mice, rats and chicks). However, we suggest a word of caution herewith, not to consider diphenhydramine a replacement for the standard antidote atropine sulfate. Diphenhydramine is merely an additional antidote that can be used in certain situations, especially in veterinary practice. Although the risk of bias outcome of the studies was mostly low, its final assessment was inconclusively unclear. This calls for strict adherence to ARRIVE guidelines in animal experimentations, especially those involved in preclinical studies. Additional studies are needed to examine the action of diphenhydramine on adverse chronic effects of ChE inhibitors.
